# Human periventricular nodular heterotopia shows several interictal epileptic patterns and hyperexcitability of neuronal firing

**DOI:** 10.3389/fneur.2022.1022768

**Published:** 2022-11-11

**Authors:** Valerio Frazzini, Stephen Whitmarsh, Katia Lehongre, Pierre Yger, Jean-Didier Lemarechal, Bertrand Mathon, Claude Adam, Dominique Hasboun, Virginie Lambrecq, Vincent Navarro

**Affiliations:** ^1^AP-HP, Pitié Salpêtrière Hospital, Epilepsy Unit and Reference Center for Rare Epilepsies, Paris, France; ^2^Sorbonne Université, Institut du Cerveau–Paris Brain Institute–ICM, Inserm, CNRS, APHP, Hôpital de la Pitié Salpêtrière, Paris, France; ^3^Institut de la Vision, INSERM UMRS 968, UPMC UM 80, Paris, France; ^4^Institut de Neurosciences des Systèmes, Aix-Marseille Université, Marseille, France; ^5^AP-HP, Pitié Salpêtrière Hospital, Department of Neurosurgery, Paris, France; ^6^AP-HP, Pitié Salpêtrière Hospital, Department de Neuroradiology, Paris, France

**Keywords:** epilepsy, periventricular nodular heterotopia, microelectrode, human, *in vivo*, interictal, seizure

## Abstract

Periventricular nodular heterotopia (PNH) is a malformation of cortical development that frequently causes drug-resistant epilepsy. The epileptogenicity of ectopic neurons in PNH as well as their role in generating interictal and ictal activity is still a matter of debate. We report the first *in vivo* microelectrode recording of heterotopic neurons in humans. Highly consistent interictal patterns (IPs) were identified within the nodules: (1) Periodic Discharges PLUS Fast activity (PD+F), (2) Sporadic discharges PLUS Fast activity (SD+F), and (3) epileptic spikes (ES). Neuronal firing rates were significantly modulated during all IPs, suggesting that multiple IPs were generated by the same local neuronal populations. Furthermore, firing rates closely followed IP morphologies. Among the different IPs, the SD+F pattern was found only in the three nodules that were actively involved in seizure generation but was never observed in the nodule that did not take part in ictal discharges. On the contrary, PD+F and ES were identified in all nodules. Units that were modulated during the IPs were also found to participate in seizures, increasing their firing rate at seizure onset and maintaining an elevated rate during the seizures. Together, nodules in PNH are highly epileptogenic and show several IPs that provide promising pathognomonic signatures of PNH. Furthermore, our results show that PNH nodules may well initiate seizures.

## Highlights

- The first *in vivo* microelectrode description of local epileptic activities in human PNH.
- Recordings revealed multiple microscopic epileptic interictal patterns.
- Firing rates of all detected units were significantly modulated during all interictal patterns.
- Seizures recruited the same units that are involved in the interictal activity.

## Introduction

Periventricular nodular heterotopia (PNH) is one of the most common types of malformation of cortical development (MCD), resulting from an abnormal neuronal migration process wherein clusters of neurons form nodular masses of gray matter close to the walls of the lateral ventricles. Stereoelectroencephalography (SEEG) is often required to identify the seizure onset zone (SOZ) and delineate the region to be potentially removed (1, 2) or partially coagulated (3). In other types of MCD, the underlying epileptogenic process can sometimes be inferred from patterns of interictal activity that are known to correlate with specific epileptogenic lesions (4, 5). For example, patients with type-IIb focal cortical dysplasia often show localized and continuous periodic spikes or poly-spikes (6, 7), while persistent and rhythmic fast-frequency discharges of very high amplitude (>100 μV) are typical of lissencephaly (8).

Recently, SEEG studies have suggested that stereotypical patterns could help in the identification of PNH. Tassi et al. (2) and Mirandola et al. (9) found an interictal pathological activity that consisted of low voltage low frequency, interrupted by frequent high-voltage spikes, followed by positive waves. These spike and wave complexes were shown to originate only from nodules and were very similar across patients and nodules. Pizzo et al. (10) also found that interictal activity in the heterotopic cortex generally displays low-amplitude activity and is further characterized by spikes and spike-and-wave complexes. The fast activity was often found as well, often superimposed on the slow wave following the spike.

Neurons within the ectopic tissue are highly disorganized (11, 12). This limits the spatial summation of electric currents measured by standard SEEG electrodes. Furthermore, it would make local field potentials (LFPs) highly susceptible to the spread of electrical activity originating in the surrounding tissue. Intracranial *micro*electrodes, due to their high impedance, record the LFPs and action potentials from a much more focal region (≈ 140 μm^3^) and are less dependent on the spatial summation of current flow (13). This spatial selectivity allows the selective measurement of ectopic neuronal behavior by permitting the evaluation of interictal patterns recorded unequivocally within nodules. To the best of our knowledge, *in vivo* recordings of single units have not yet been reported, and neither has interictal activity in PNH been measured with the spatial selectivity provided by microelectrodes. The investigation of the behavior of single units during interictal activity will, therefore, shed new light on the epileptogenicity of ectopic neurons in PNH and their role in generating interictal activity.

The question of whether nodules are typically part of the SOZ in PNH has been widely addressed in previous SEEG studies, showing that PNH involves complex, patient-specific epileptic networks that include nodules, as well as the overlying cortex and mesial temporal structures when adjacent to nodules (1, 2, 9, 10, 14–17). The current study will further substantiate the involvement of ectopic neurons in seizures by investigating the involvement of ectopic neurons during both interictal activity and seizures.

Most of our knowledge of PNH on the cellular level comes from animal models. Preclinical PNH animal models (methylazoxymethanol rat) found abnormal bursting-like properties and prolonged tonic firing in PNH neurons (18), together with a lack of functional A-type Kv4.2 potassium channels (19). These findings suggest increased excitability and decreased seizure thresholds in PNH neurons. In line with the increased excitability in PNH, *in vitro* studies found a significant prolongation of GABAergic inhibitory postsynaptic potentials in heterotopic neurons (20, 21) and reduced NR2A and NR2B subunit expression in methylazoxymethanol rat models, as well as in resected human PNH tissue (22). These findings suggest a compensating drive against the otherwise hyperexcitability of the pathological network of PNH.

The current study is the first *in vivo* recording of extracellular action potentials in awake behaving human patients, allowing analyses of the firing behavior of ectopic neurons during interictal and ictal activities. Specifically, we (i) analyzed the activity of heterotopic neurons during interictal patterns (IPs), (ii) investigated whether interictal patterns previously described on macroelectrodes are consistent with those found on the microscopic level, and iii) explored whether neurons that respond to interictal activity are also involved in seizures. For this purpose, the IPs were described in microelectrode recordings from four PNH nodules in three patients. Single units were extracted using spike-sorting algorithms and their firing rate was time-locked and correlated with the IPs. We expected to find (i) consistent IPs within and between all four nodules, consistent with those described on macroelectrodes, (ii) hyper-excitable neuronal firing during the IPs, shown by a significant proportion of cells responding to interictal activity in a time-locked manner, and (iii) that units that respond to interictal activity are recruited during seizures.

## Materials and methods

### Patients

Three patients suffering from pharmacoresistant focal epilepsy due to PNH were included (refer to Table 1). Patients received a full presurgical evaluation in the Epileptology Unit of the Pitié-Salpêtrière Hospital. All patients underwent 27-electrode long-term video-EEG recordings (Micromed System, Italy). The presurgical evaluation also included a structural 3T epilepsy-oriented brain MRI protocol, interictal 18FDG PET, and ictal (SISCOM) Tc-99m-HMPAO SPECT analysis. In Patient 1, MRI revealed a heterotopic nodule on the temporal horn of the right ventricle, while Patient 2 presented with bilateral nodules predominantly in the posterior regions. In Patient 3, periventricular nodules were located in the posterior part of the right temporal lobe. In Patients 1 and 3, periventricular nodules were also associated with other anatomical abnormalities, including subcortical nodules (Patients 1 and 3), dysplastic overlying cortex (Patient 1), and polymicrogyric overlying temporal cortex (Patient 3). Data from Patient 1 were analyzed for 20.6 consecutive hours. Data from Patient 2, due to an abundance of interictal activity, were analyzed for 6 h. In Patient 3, interictal activity was analyzed for 17.5 consecutive hours (Table 2).

**Table 1:** Clinical summary.

**Age at 1st seizure (Sex)**	**Type**	**Epilepsy duration until sEEG**	**IEDs (scalp EEG)**	**Seizures** **(Scalp EEG)**	**SOZ defined by sEEG and % from the total number of seizures**	**MRI**	**PET**	**SPECT**	**Med**.
*20 (M)*	FIAS FBTCS	11 yrs	Right anterior temporal regions	Right temporal region, spread to bilateral frontal regions	89% the nodular lesion (*n* = 40). 11% (*n* = 5) postero-inferior cortical malformations close to the right hippocampal formation	PNH in the temporal horn of the right ventricle. Adjacent SNH and dysplastic overlying cortex	Hypermetabolism PNH	-	LTG CBZ LCM CBZ
*25 (F)*	FIAS FBTCS	5 yrs	Left temporo- occipital region	Left posterior temporal region	74 % (*n* = 14) left PNH. 25% (*n* = 5) diffuse origin	Bilateral posterior PNH. No associated cortical abnormalities	No metabolic changes of the PNH	Hyperperfusion of the cortical structures adjacent to the nodules. No evident metabolic modification in PNH	LTG
*14 (F)*	FIAS FBTCS	16 yrs	Right temporal or fronto-temporal region. Focal fast oscillations (20–24 Hz) in the right temporal lobe	Right temporal region	44% (*n* = 20) nodules. 38% (*n* = 17) right posterior parahippocampal region. 18% (*n* = 8) diffuse origin	Right temporo-posterior PNH Temporal SNH and PMG	Isometabolism of the PNH, PMG and adjacent cortices	Ictal hyperperfusion of PNH, PMG and adjacent and right temporo-polar cortices	LTG ZNS LCM

AED, Antiepileptic drugs; IEDs, Interictal Epileptiform discharges; FIAS, Focal Impaired Awareness Seizure; FBTCS, Focal to Bilateral Tonic-Clonic Seizures; PNH, Periventricular Nodular Heterotopia; PMG, Polymicrogyria; SNH, Subcortical Nodular Heterotopia; CBZ, Carbamazepine; LCM, Lacosamide; LTG, Lamotrigine; ZNS, Zonisamide.

**Table 2:** Localization of electrode contacts.

		**Macro contacts**							
**N**	**Micro**	**1**	**2**	**3**	**4**	**5**	**6**	**7**	**8**
1 (R)	GM PNH	GM PNH	GM PNH *lat*	GM STS	GM STS	GM STS	GM SMG	GM SMG	EC
2 (R)	GM PNH	GM PNH	GM PNH *lat*	WM	GM STG	GM STS *T2*	GM STS *T2*	GM *T2*	GM *T2*
3 (L)	GM PNH	GM PNH *im*	GM PNH *im*	LTO	LTO *T3*	WM	WM	GM/WM *T3*	GM *T3*
4 (R)	GM PNH)	GM PNH	GM PNH	WM	WM	GM STS	GM/WM T1	GM T1	EC

**N**, Nodule; **L**, Left; **R**, Right hemisphere; **PNH**, Periventricular Heterotopic Nodule; **GM**, Gray matter; **WM**, White matter; **lat**, lateral; **im**, inferior medial; **LTO**, Lateral Temporo-Occipital Sulcus; **SMG**, SupraMarginal Gyrus; **STS**, Superior Temporal Sulcus; **T1**, Superior Temporal Gyrus; **T2**, Middle Temporal Gyrus; **T3**, Inferior Temporal Gyrus; **EC**, Extra Cerebral.

### SEEG recording

All intracranial SEEG procedures were performed according to clinical practice and the epilepsy features of the patients. In addition to macroelectrodes, Behnke–Fried-type (23) macro–microelectrodes (AdTech^®^) were inserted in nodules (Table 2). Signals from macro- and microelectrodes were continuously and synchronously recorded at 4 and 32 kHz, respectively, using a hardware filter at 0.01Hz (Atlas Recording System; NeuraLynx, Tucson, AZ, USA). Post-implantation electrode locations were based on a pre-implant 3T 3D-MRI, post-implant 1.5T 3D-MRI, and CT scan, integrated using the Epiloc toolbox, an in-house developed plugin for the 3D-Slicer visualization software (24, 25). Patients gave written informed consent (project C11-16 conducted by INSERM and approved by the local ethic committee, CPP Paris VI).

### Visual analysis of micro-LFP patterns

Pathological activities were visually identified on microelectrode LFP signals, according to traditional morphological characteristics used in clinical practice, and then classified into different interictal patterns (IPs). Criteria for each pattern were then used for a complete manual scoring of continuous data recorded on the first day after implantation. The annotations were performed on the first day after the implantation when the quality of the microelectrode signal is typically the highest. For each nodule, the annotations of the IPs were based on the LFP from the same microwire, selected for the clearest morphology, using software developed in-house (MUSE). We describe our classification according to standard guidelines (26, 27). Albeit designed for the critical care unit, such standard terminology improves clarity in communication, and to the best of our knowledge, no other such standard exists. For periodic patterns, only sequences with at least three consecutive slow waves were considered. On a subset of these patterns, a detailed analysis of their periodicity was performed, by calculating the percentage of deflections that deviated <25% from the average period within each pattern (26, 27).

### Seizure onset zone based on video-SEEG

The seizure onset zone of each patient was determined as part of the standard clinical procedure, involving visual annotations and analyses of the entire video-SEEG recordings by trained epileptologists (VF, CA, and VN). In addition, for every seizure during the entire recording, the SOZ was scored as either nodular, non-nodular (cortical or hippocampal), or diffuse, resulting in a percentage of nodular seizures per patient. Representative examples of SEEG recording during seizures are reported in Supplementary Figure S3.

### Time-locked and time–frequency analysis

Analyses were performed with a combination of FieldTrip (28) and custom MATLAB scripts (The MathWorks Inc., Natick, Massachusetts). Manual annotations of each pattern, in each nodule, were temporally aligned by maximizing the cross correlation between electrode time courses. Data were then down-sampled to 1 kHz. The average LFPs were extracted based on the aligned time courses. Time–frequency representations (TFRs) were calculated using a Hanning taper applied to a sliding time window in steps of 5 ms. The time window was 200 ms for epileptic spikes and 400 ms for all other (longer) patterns. The average power in 60–200 Hz was expressed in percentage change relative to the baseline period that was set at −1s to −0.5 s for epileptic spikes and −2 s to −1 s relative to pattern onset (annotation) for the other patterns.

### Spike sorting

All spikes occurring during manually annotated artifact periods were ignored. After selecting electrodes that showed multi-unit activity (MUA), data were temporally whitened, and spikes were automatically detected at 6 (Nodules 1, 2, and 4) or 5.5 (Nodule 3) median absolute deviations of high-pass filtered (>300 Hz) data. A combination of density-based clustering and template matching algorithms was used to automatically cluster the detected spikes [*Spyking Circus*, (29)]. Clusters were visually merged when considered similar, based on the spike morphology, firing rate, amplitude, and cross correlation. Clusters were evaluated as to whether they reflected putative single-unit activity (SUA) or multi-unit activity, based on the inter-spike interval (ISI), the percentage of refractory period violation (RPV = ISI < 2 ms), and spike morphology.

### Spike time analyses

Spike times were epoched and time-locked according to the aligned annotations. The average spike rates were calculated continuously at 1,000 Hz, using a Gaussian smoothing kernel of 10 ms for epileptic spikes and 50 ms for the longer patterns to better capture the slower modulations of firing rate. Correlations were calculated between the average time-locked spike rates of each unit and the average time-locked LFP of every pattern. Finally, the spike rates of each trial were binned into 100 bins for statistical analyses. To determine the resting behavior of detected units, data were first to split into 10-s time periods, excluding time periods that overlapped with IED or seizure occurrence. The mean firing rate and coefficient of variation (30) were calculated over the remaining time periods. Spike trains were time-locked to the manually annotated onset of every seizure.

### Statistical analysis

To control for multiple comparisons and non-normal distributions of firing rates, we performed nonparametric cluster-based permutation tests to determine time periods where firing rates changed significantly from baseline (31). A threshold of *p* < 0.01 (first-level *t*-test) was used to determine contiguous clusters, after which a threshold of *p* < 0.05 (one-sided correction) determined whether the clusters (the sum of *t*-values) could be explained under permutation (Monte Carlo *n* = 10.000). Due to the limited number of seizures, no frequentist statistics were performed on firing rates during seizures. However, the raster plots were created, allowing qualitative observations on whether units involved in the IPs were also recruited during seizure onset.

### Data availability statement

All scripts are made available on GitHub: https://github.com/stephenwhitmarsh/EpiCode/PNH. Anonymous data can be made available on reasonable request.

## Results

### Interictal epileptic LFP activities revealed by microelectrodes in PNH

**Figure 1:**
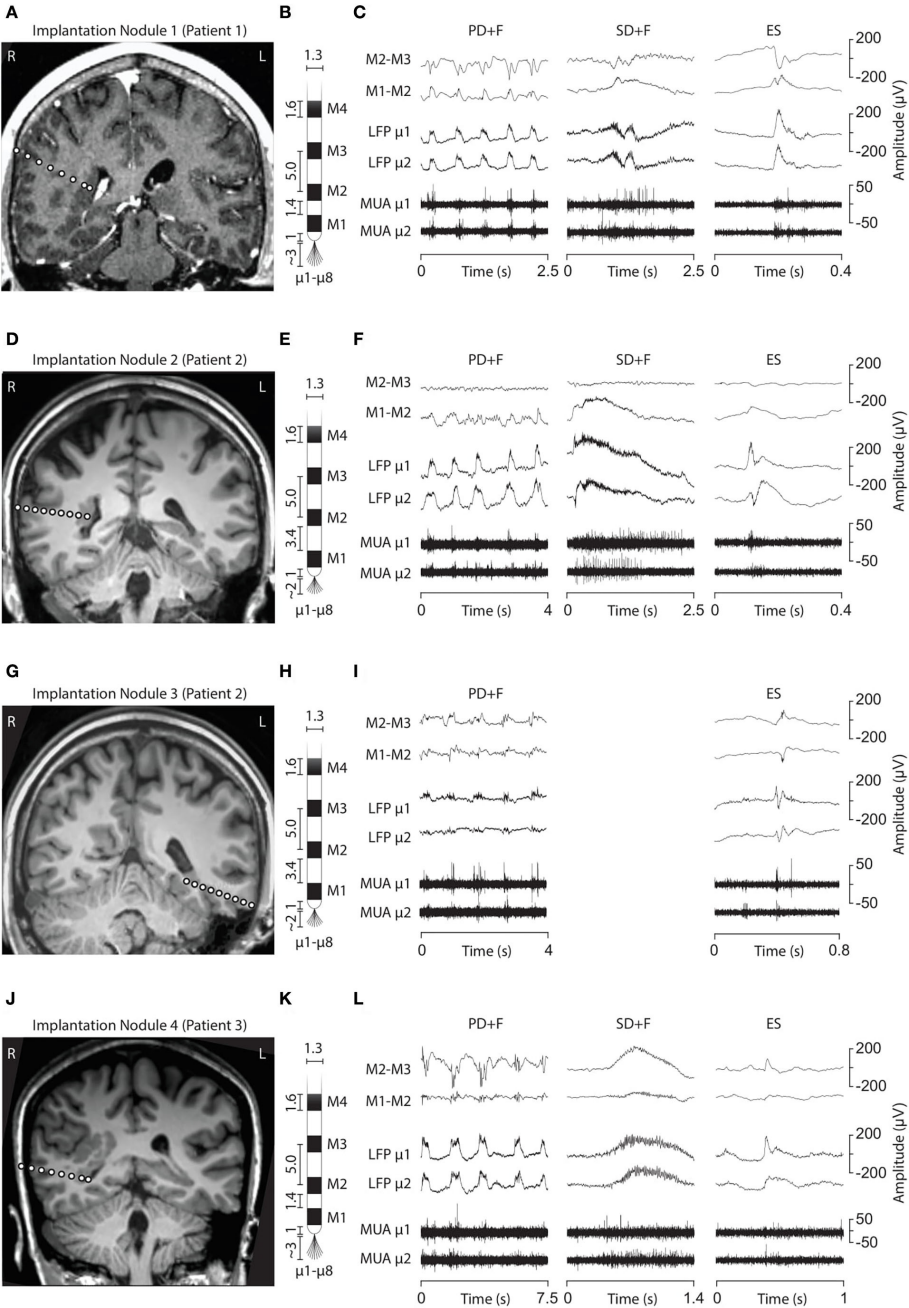
*Electrode implantation into the PNH nodules and interictal LFP patterns*. **(A, D, G, J)** Brain MRI showing the electrode trajectories, exploring the four analyzed nodules (A, Patient 1, Nodule 1; D and G, Patient 2, Nodules 2 and 3; J, Patient 3, Nodule 4). **(B, E, H, K)** Schematic representation of the macro–microelectrodes (M1–M4: macroelectrode contacts; μ1–μ8: microelectrodes). All the geometrical features of the electrodes are expressed in millimeters. Macroelectrode recordings **(C, F, I, L)** are shown in a bipolar montage. Three LFP patterns were recorded: periodic discharge plus fast activity (PD+F), sporadic discharges plus fast activity (SD+F), and epileptic spikes (ES). These patterns were apparent on microelectrodes (LFP μ1 and μ2) and to a smaller degree also on macroelectrodes (M1–M2). LFP patterns were associated with MUA recorded on the microelectrodes.

Three interictal patterns (IPs) were consistently detected by microelectrodes in the nodules (Figure 1).

#### Periodic discharges PLUS fast activity

**Figure 2:**
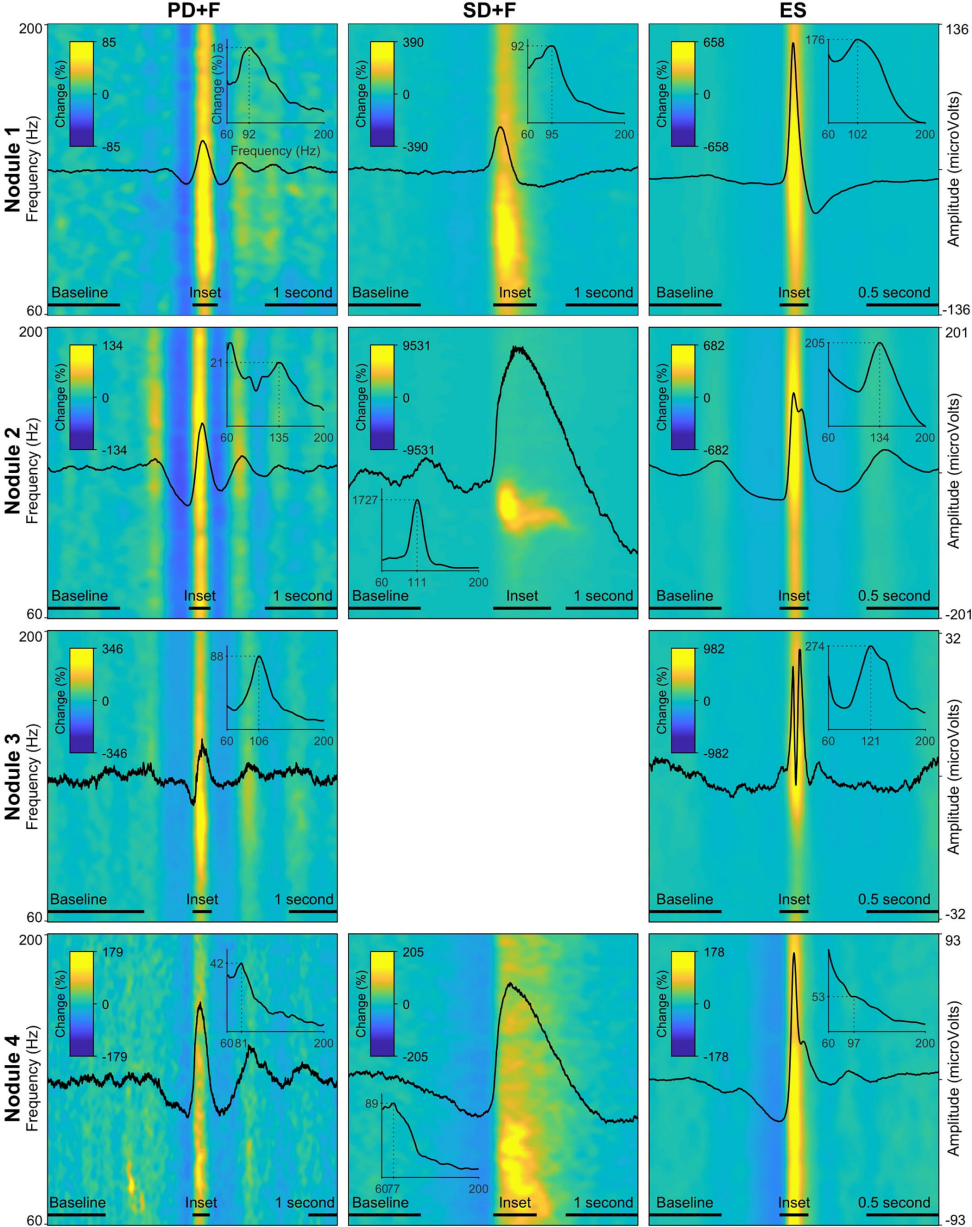
Local field potentials (LFP) and time–frequency spectra of the interictal patterns. Each row represents one nodule, with every panel/column an interictal pattern, indicated by the panel title. The amplitude of the LFP is scaled per nodule and displayed on the far-right y-axis of every row. The average time–frequency representations (60 Hz−200 Hz) are expressed in percentage change from the baseline period, indicated by the color bar in every panel. Insets show the average power spectra during the peak activity of every pattern, indicated by a horizontal bar above the x-axis. The dotted lines in the inset show peak frequency and % power increased versus baseline. Note that due to the fact that these values are averaged over a time segment, the percentage change is lower than indicated in the time–frequency plots.

We identified sequences of periodic slow waves, with superimposed low-voltage fast activity. They were defined as Periodic Discharges PLUS Fast activity (PD+F) and were identified in all nodules (Figure 1). These patterns were clearly detectable on microelectrodes and to a smaller degree on the adjacent macroelectrode contacts (Figures 1C,F,L, left panel). PD+F appeared as periodic bursts of fast activity on the closest macro-contacts (M1–M2). In Nodule 3, periodic fast activity discharges (PF) consisted of periodic fast activity (PF; Figure 1I, left panel). The PD+F patterns showed clear periodicity in the average LFP time course (Figure 2). Consistent with the observation of low-voltage fast activity in the single-trial time courses, the periodicity was also clearly visible in the frequency domain, with peak increases at 92 Hz, 135 Hz, 106 Hz, and 81 Hz for Nodules 1, 2, 3, and 4, respectively (Figure 2). A more detailed annotation within a subset of periodic patterns showed that the majority of deflections within a single train varied <25% of the mean interval between deflections (Table 3), conforming to the definition of *periodicity* (26, 27).

**Table 3:** Descriptive statistics of (inter)ictal activity for each nodule.

		**PD**+**F**						**SD**+**F**		**Epileptic Spike**		**Seizure**
**N**	**t**	***n***	**λ**	**xΔt**	**ρΔt**	**Periodicity**		***n***	**λ**	***n***	**λ**	***n***
1	20.6	1,485	291	511	18.7	124/150	83%	950	302	3,674	590	6
2	6.0	950	1,445	574	20.6	93/117	80%	89	4,499	2,685	494	0
3	6.0	518	1,289	1,074	17.6	116/128	91%			392	373	0
4	17.5	231	4,807	1,723	19.6	62/72	86%	507	1,169	2,504	361	8

N, Nodule; t, Total duration of time analyzed (hrs.); PD+F, Periodic Discharges PLUS Fast activity; SD+F, Sporadic Discharge PLUS Fast activity; ES, Epileptic Spike; n, number of (inter)ictal events; λ, Mode of time between events (ms); xΔt, Average time between periods (ms); ρΔt, Standard deviation of time between periods (ms); Periodicity, ratio & percentage of periods that deviate <25% of the average time between periods.

#### Sporadic discharges PLUS fast activity

We also identified large slow waves with superimposed and prolonged low-voltage fast activity in Nodules 1, 3, and 4 (Figures 1C,F,L, middle panels). We termed this pattern Sporadic discharges PLUS Fast activity (SD+F). SD+F were also detectable on the macroelectrodes located in the nodules, despite smaller amplitudes (M1–M2 and M2–M3). These events were sporadic, with no tendency to further ictal organization. In Nodule 1, SD+F consisted of fast activity superimposed on a polyphasic slow deflection of 0.5–1 s (Figure 1C, middle panel), while in Nodules 2 and 4, fast activity occurred at the onset of a sharply appearing monophasic slow deflection of 2–4 s (Figures 1F,L, middle panel). Time–frequency analysis showed that in Nodule 1, power increased at 96 Hz for ≈ 0.5 s (Figure 2, top middle panel). Nodule 2 showed a narrow-band increase in power at 111 Hz for at least 1 s (Figure 2, center middle panel). In Nodule 4, SD+F consisted of an increase in power in a broad frequency range, peaking at 77 Hz and lasting for 1–2 s (Figure 2, lower center panel).

#### Epileptic spikes

Epileptic spikes (ES) were identified in all four nodules on both the micro- and adjacent macroelectrodes contacts (Figures 1, 2). Nodule 1 showed isolated sharp monophasic waves (Figure 1C, inset on the right). In Nodule 2 (Figure 1F), spikes were generally followed, and often preceded, by a slow wave, which was also apparent in the average time-locked LFP. In Nodule 3 (Figure 1I), spikes were characterized by a low-amplitude di- or triphasic wave. In Nodule 4, spikes appeared as sharp monophasic waves often following a slow wave (Figure 1L, right panel). The low-voltage fast activity was often superimposed on ES, with a mean peak at 102, 134, 121, and 97 Hz, respectively. These ES were clearly visible also on macroelectrodes (Figures 1C,F,I,L).

Patterns occurred frequently, often within seconds of each other. Table 3 reports the mode (λ) of each pattern, i.e., the most frequent time interval between events. The IPs correlated strongest with the LFPs of the closest macroelectrode contacts (M1–M2), after which the correlation reversed, showing that the IPs were located within the PNH (Supplementary Figure S2).

### All neurons are recruited by interictal patterns

**Figure 3:**
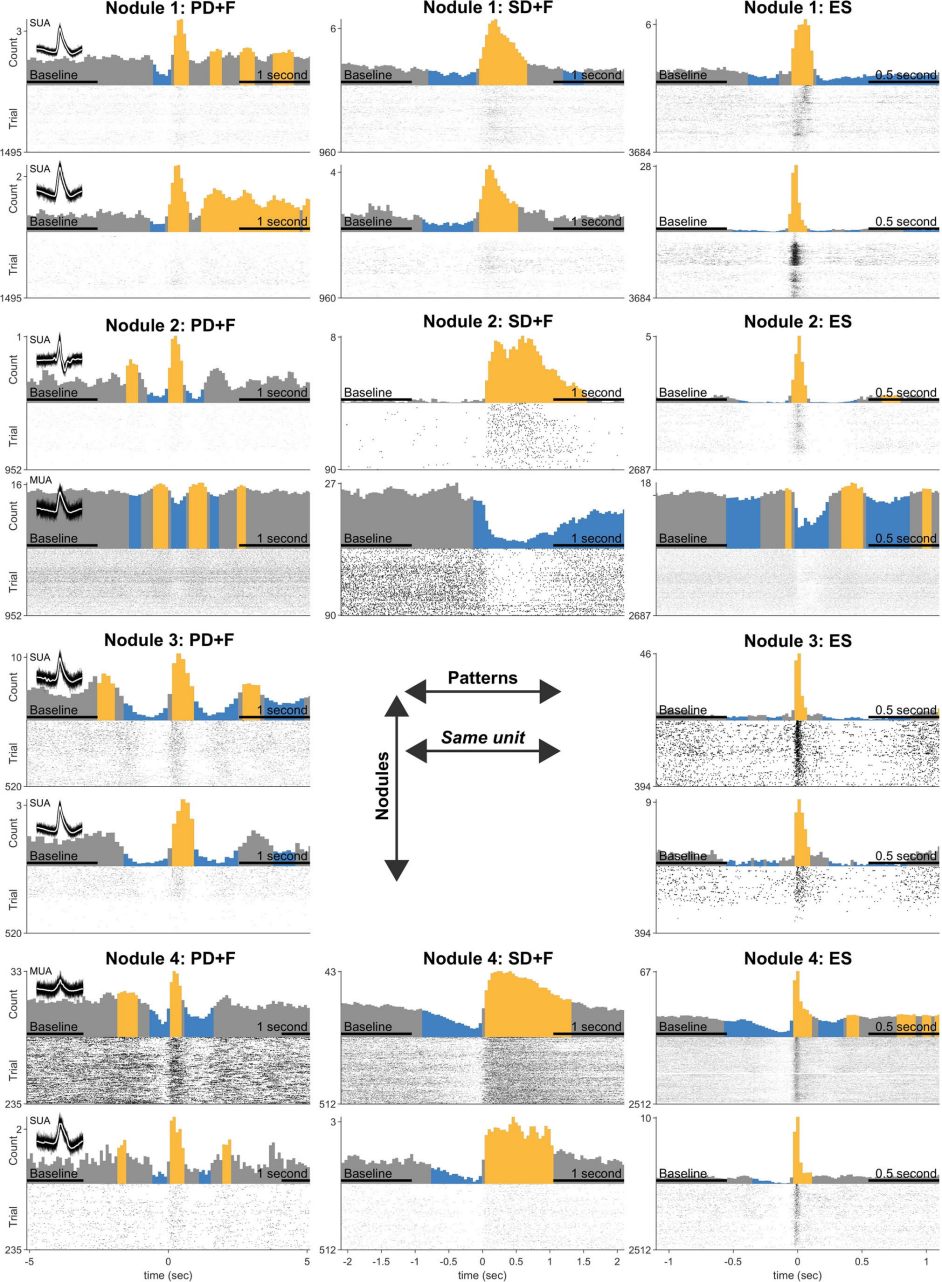
Statistical analysis of firing rate responses during the IPs. Each row represents the firing responses of one unit, shown with a firing rate histogram centered on the onset of IP, above the respective raster plot. The yellow color indicates significant increases and the blue color decreases compared with the average firing rate during baseline (indicated in the x-axis). Two units are shown for every nodule (three patients). Panel titles indicate an interictal pattern. Note that each unit (row) shows significant modulation in every pattern. Inset shows 100 examples of action potential spikes, overlaid with their average spike waveform in white.

In addition to the LFP signals, microelectrodes recorded action potentials [multi-unit activity (MUA)] in 24 microwires, located in periventricular nodules (Nodule 1: *n* = 5, Nodule 2: *n* = 7, Nodule 3: *n* = 8, Nodule 4: *n* = 4). Spike clustering resulted in 39 units of which 18 (55%) were classified as single units (Table 4). All units were shown to significantly modulate their firing rates in response to all IPs. The firing rate increased up to 472% for PD+F, 10,234% for SD+F, and 2138% for ES (Table 4). Most of the units also showed brief episodes of decreased firing rate surrounding the IPs, often by ≈100%, i.e., silence (Table 4 and Figure 3). Especially during ES, firing rates strongly decreased in all units within ≈500 ms surrounding the discharge. Furthermore, correlations between firing rates and LFPs showed that the modulation of firing rates was highly consistent with the timing and shape of the IPs in all units (Table 4). A massive increase in firing rate was shown for the very short duration of the ES, while SD+F was associated with a more prolonged increase in firing rate (Figure 3). During PD+F, firing rates showed regular and alternating periods of increases and decreases. Interestingly, two clusters (one SUA and one MUA) significantly modulated their firing rate inversely with respect to the IPs; they decreased their firing rate during interictal LFP activities (Table 4, Figure 3: 4th row/neuron).

**Table 4:** Unit statistics.

				**PD**+**F**			**SD**+**F**			**ES**			**Baseline**		
**U**	**Nodule**	**S/MU**	**RPV**	**↑**	**↓**	**ρ**	**↑**	**↓**	**ρ**	**↑**	**↓**	**ρ**	**FR**	**Amp**	**CV2**
1	1	SUA	0.5	154	20	0.88	253	22	0.49	331	22	0.72	4.1	71.3	1.21
2			0.3	250	*n.s*.	0.59	472	52	0.74	426	61	0.24	15.2	115.0	1.19
3			1.7	124	16	0.89	227	21	0.54	261	17	0.76	4.1	49.1	1.18
4			0.7	154	38	0.77	257	33	0.63	1,971	31	0.80	26.8	47.0	1.24
5			0.3	321	25	0.75	379	28	0.61	263	26	0.61	8.2	170.1	1.24
6		MUA	1.3	171	29	0.9	256	14	0.53	292	21	0.81	3.9	38.0	1.15
7			1.6	93	10	0.78	239	30	0.55	1,098	17	0.91	3.8	28.3	1.17
8			1.9	352	53	0.72	454	52	0.59	441	56	0.30	25.5	67.5	1.18
9			3.0	121	15	0.90	231	12	0.53	366	14	0.84	5.3	30.9	1.16
10	2	SUA	0.3	192	35	0.51	460	49	0.27	597	21	0.50	2.6	55.4	1.02
11			0.2	199	40	0.74	3,846	97	0.87	1,325	48	0.75	2.3	69.6	1.27
12			1.1	*n.s*.	13	0.31	*n.s*.	36	0.28	79	15	0.71	6.5	29.8	1.04
13			0.1	*n.s*.	14	**− 0.26**	*n.s*.	68	**− 0.39**	36	20	**− 0.31**	3.8	53.2	0.86
14		MUA	0.8	179	19	0.58	990	38	0.49	794	30	0.56	3.4	43.3	1.00
15			2.4	260	36	0.69	3,193	99	0.72	962	41	0.75	5.5	29.5	1.15
16			0.8	247	40	0.51	10,234	*n.s*.	*n.s*.	905	23	0.76	3.6	41.5	1.17
17			1.3	125	25	0.41	*n.s*.	86	0.08	363	29	0.67	41.8	65.9	1.09
18			4.0	161	19	0.70	1,216	50	0.85	769	25	0.63	7.3	30.3	1.04
19			1.3	15	9	**−0.79**	*n.s*.	15	**−0.62**	19	16	**−0.62**	14.1	29.5	0.77
20			2.0	191	18	0.70	1,480	51	0.68	621	15	0.83	3.7	30.7	1.17
21			2.4	255	31	0.80	4,718	*n.s*.	0.88	1,181	31	0.75	12.2	55.7	1.27
22			3.0	146	23	0.71	1,381	*n.s*.	0.84	522	19	0.72	8.8	32.2	1.01
23	3	SUA	2.2	123	18	0.63				847	36	0.57	4.0	39.2	1.16
24			0.2	290	60	0.55				*n.s*.	100	0.53	5.8	80.2	1.24
25			0.3	426	54	0.59				2,138	99	0.56	20.8	61.6	1.17
26			0.5	168	60	0.58				1,087	66	0.45	2.2	33.7	1.18
27			0.7	143	35	0.72				484	61	0.75	2.2	71.6	1.19
28			0.5	368	35	0.69				819	76	0.62	1.5	40.4	1.29
29			0.1	343	37	0.51				1,211	80	0.62	1.5	51.6	1.18
30		MUA	1.0	153	52	0.56				1,398	41	0.51	2.2	33.5	1.18
31			0.6	117	46	0.54				655	58	0.52	1.4	31.2	1.12
32			2.1	176	51	0.70				625	45	0.53	3.6	19.7	1.25
33			0.3	472	48	0.58				1,571	50	0.54	3.7	32.9	1.22
34	4	SUA	0.8	*n.s*.	33	0.45	241	32	0.81	*n.s*.	17	0.22	2.8	87.1	1.04
35			0.5	126	47	0.70	182	31	0.84	876	25	0.79	2.1	40.1	1.19
36		MUA	4.6	54	27	0.77	109	16	0.89	231	11	0.92	16.4	23.8	0.76
37			6.6	59	17	0.77	112	9	0.89	184	9	0.90	21.7	20.3	0.69
38			3.6	78	22	0.84	120	12	0.89	366	9	0.89	11.3	18.1	0.83
39			1.7	*n.s*.	54	0.51	155	42	0.82	323	56	0.73	2.3	59.0	1.12

U, unit; S/MU, Single Unit Activity (SUA) or Multi Unit Activity (MUA); RPV, Refractory Period Violation (% > 2 ms); PD+F, Periodic Discharge PLUS Fast activity; SD+F, Sporadic Discharge PLUS Fast activity; ES, Epileptic Spike; Values under up (↑) and down (↓) arrows show the maximum percentage increase or decrease in significant clusters; ρ, pearson correlation between peri-stimulus time histogram and LFP; Negative correlations in bold font; Only significant values (p < 0.01) are shown; FR, Amp and CV2, Firing rate, average spike waveform amplitude and Coefficient of Variation, based on 10 second intervals without overlap with IEDs.

### Nodules are involved in seizure initiation

The continuous 3-week SEEG recordings allowed us to describe the complex ictogenesis of patients with PNH. In all patients, seizures started predominantly in the PNH (from 42 to 90% of the total recorded seizures; see Table 1). In Patient 1, seizures from nodules were characterized by the appearance of a slow deflection overlapping with fast rhythms between 115 and 160 Hz (Supplementary Figure S3). Seizures started from the deep contacts of the two macroelectrodes, located in the posterior part of the PNH (Table 1), strongly implicating the PNH as the SOZ. In Patient 2, seizures were characterized by fast rhythms (above 270 Hz), starting in the contacts located within the anterior part of the left nodule, with a very fast involvement of the posterior part of homolateral PNH and the adjacent temporal cortex. The SOZ was, thus, represented by a complex network involving both the left PNH and the adjacent cortex. In Patient 2, the right nodule was not part of the SOZ but did eventually take part in the later stages of seizure development. In Patient 3, focal seizures from the nodule started as a slow deflection with superimposed fast rhythms visible on the deepest contacts located in the most inferior and posterior parts of the nodular lesions. Because of the extent of the pathological lesions and the complexity of the epileptic networks, none of the included patients was selected for traditional surgical resection procedures. Patient 1 underwent laser interstitial thermal therapy (LITT) intervention, targeting the heterotopic nodule on the temporal horn of the right ventricle, identified as the main site of seizure generation during the SEEG recordings. At the time of writing, the patient did not present any seizures after the LITT procedure (2 months). A longer follow-up is needed to evaluate post-surgery prognosis.

### Units participating in the IPs are also recruited during seizures

**Figure 4:**
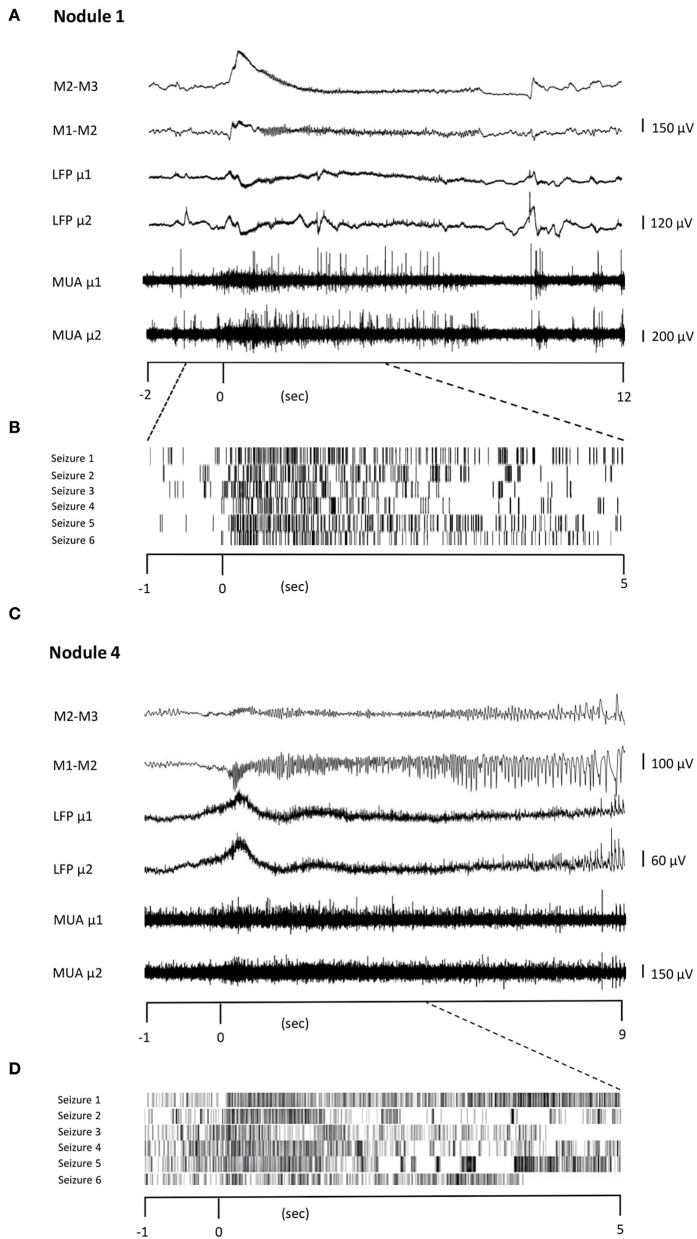
Unit firing behavior during seizures. Two representative seizures originating from Nodule 1 **(A)** to Nodule 4 **(C)**, respectively. The ictal activity from two adjacent contacts of the macroelectrode (M1–M2, M2–M3, bipolar montage) is reported together with that obtained from two different microelectrodes (μ1 and μ2). The microelectrode activity is then shown as LFP and MUA activity. TRaster plot, time-locked to seizure onset, for Nodule 1 **(B)** and Nodule 4 **(D)**. In the raster plot, different shades of gray denote different units. Each line of the raster plot shows the unit's behavior during a seizure. The first line in the raster plot corresponds to the seizure shown above. Note that the units are the same as those analyzed for IEDs, here showing that they are also recruited during seizures.

In Patients 1 and 3, seizures were recorded during the periods in which MUA could be observed, allowing the observation of firing rate modulation during seizure initiation. In Patient 2, seizures occurred late during the continuous recordings, during a period where no MUA were present anymore. In Patient 1, all six seizures started in the nodule (Nodule 1). In Patient 3, six seizures started from the nodule (Nodule 4), one seizure had a diffuse onset, and one seizure could not be analyzed due to artifacts. In total, unit firing times were recovered during 12 seizures. The recovered units were the same as those analyzed for the IPs. Although the relatively small number of observations precludes statistical tests, units were shown to increase their firing rate in time with seizure onset and maintain an elevated rate for its duration (Figure 4). This strongly suggests that units take part not only in different types of interictal activities but are also recruited during seizure onset.

## Discussion

Two novel interictal patterns (IPs) were identified in all four PNH nodules: Periodic Discharges PLUS Fast activity (PD+F) and Sporadic Discharges PLUS Fast Activity (SD+F). Epileptic spikes (ES) were also shown to occur often and consistently in all four nodules. We showed that the IPs originated from the nodules and not from the adjacent cortex. First, the microelectrode locations were validated by neuroimaging to be located within the PNH. Second, due to their small size and high impedance, microelectrodes record highly localized activity in the order of 1 mm^3^ (32). Third, the LFPs only correlated with neighboring macro-contacts that were also located within the PNH (Supplementary Figure S2).

Microelectrode also allowed the recording of multi-unit activity within PNH. We found that all spontaneously active neurons modulated their firing rates in accordance with the LFPs of all IPs. Such coincidence of neocortical neuronal discharge with IEDs recorded with surface EEG, i.e., “Positive” (33) or “involved” neurons (34), was suggested to be an indicator of the intensity of epileptic activity (35). Understanding the relationships between single-unit activity and IEDs in the human brain has proven to be complex, however. Previous microelectrode studies found a strong heterogeneity in neuronal firing rates during cortical (36, 37) as well as during mesial temporal interictal discharges (38, 39), but none have, as of yet, reported on PNH. Some studies showed that only a subset of units increases its firing rate during the fast component of the epileptic spike (36–38, 40, 41). More consistent behavior was found during the slow component of the ictal discharge, during which the firing rate is demising, independent of their cortical or limbic origin (37–39, 41). In the current study, both during the fast component of the epileptic spikes and during the slow components of both the epileptic spikes and novel periodic patterns, all the detected units significantly modulated their firing rates (Figure 3), with the majority of units increasing during fast activity and decreasing during slow activity (Table 4). Together, our results point to a highly pathological and reactive neuronal organization in PNH. This is in line with data obtained from rodent models, where PNH neurons showed aberrant cellular expression of ionic channels and thus cellular hyper-excitability (18–20, 22). Indeed, heterotopic nodules show only a rudimentary laminar pattern and a lower degree of organization (11, 12).

High-frequency oscillations (HFOs) were shown to be superimposed onto all three IPs. We found narrow-band increases in power in the high-gamma and ripple domain during SD+F (96, 111, and 77 Hz, in Nodules 1, 2, and 4, respectively), ES (102, 134, 121, and 97 Hz, in Nodules 1 to 4, respectively), and PD+F (92, 135, 106, and 81 Hz, in Nodules 1 to 4, respectively). Ripples (90–250 Hz) have been found in PNH nodules before, independent of whether the nodule was part of the SOZ (10, 42). In patients with PNH, fast ripple (250–500 Hz) generation was identified in normotopic SOZ (15). Interestingly, in our findings, the SD+FA pattern was found only in the three nodules that were actively involved in seizure generation (Nodules 1, 2, and 4), but was never observed in the nodule that did not take part in ictal discharges (Nodule 3).

The role of PNH nodules in the initiation of seizures is still a matter of debate. Presurgical investigations in patients with PNH suggest that periventricular heterotopia might best be considered as part of a complex pathological network that engages both nodules and cortical structures, resulting in seizures that might start in PNH, in the overlying cortex, or in both (1, 2, 9, 10, 14–17, 43). Studies on animal models of PNH showed that heterotopic nodules are reciprocally connected with both the neocortical structures and the hippocampus (18, 44, 45). Indeed, *in vitro* as well as *in vivo* electrophysiological recordings in MAM rats also demonstrated that PNH can independently generate interictal epileptiform activity despite not being part of the SOZ (46, 47).

One limitation of this study is the small number of investigated patients. This is due to the rarity of PNH and the limited number of patients with PNH who are good candidates for surgery and therefore for intracerebral investigations. Finally, the placement of microelectrodes together with macroelectrodes into heterotopic nodules is not always possible for anatomical reasons. This limitation may explain some differences in SOZ localization compared to other studies, where the heterotopic tissue was found to be the SOZ in only 20% of patients with PNH (9, 10). On the other hand, all heterotopic nodules showed ES and PD+F interictal patterns, regardless of whether the nodules were at the origin of seizures suggesting a common microscopic organization of the interictal epileptic activity in heterotopic neurons. These interictal patterns appear reproducible and robust despite the limited number of patients in our study. As mentioned earlier, one interictal pattern (SD+FA) was found only in the three nodules that were actively involved in seizure generation (Nodules 1, 2, and 4), while it was never observed in the nodule that did not take part in ictal discharges (Nodule 3). This finding might serve as a potential signature of the SOZ in PNH. Interestingly, our results indicate that the units that were active during the interictal activity were also involved in ictal activity, extending previous findings showing a varied response of neurons during seizures (48–51). Unfortunately, in the bilaterally implanted patient, MUAs were not present anymore at the time of seizures, thus, a comparison between ictogenic nodules and non-ictogenic nodules could not be made.

Behnke–Fried-type macro–microelectrodes are only able to extrude microwires at the deeper extremity of the electrodes, making the lateral or adjacent neocortex inaccessible. The ability to record microelectrodes signals simultaneously from heterotopic and adjacent normotopic cortices (e.g., lateral or basolateral temporal cortex) will add new valuable information on the epileptic network associated with PNH lesions.

To conclude, this study presents the first *in vivo* microscopic description of the behavior of ectopic neurons during several interictal epileptic patterns. Compared with other epileptic tissues, PNH presented as a relatively clearly defined structure that was easily targeted for implantation with microelectrodes. Our study indicates consistent pathological, hyper-excitable activity within heterotopic nodules. On the level of the LFP, sporadic and periodic interictal patterns with superimposed fast activity might provide a pathognomonic signature of PNH. Future research will have to determine whether these IPs are specific to PNH, or might be found in other types of cortical malformations as well.

## Data availability statement

The raw data supporting the conclusions of this article will be made available by the authors, without undue reservation.

## Ethics statement

The studies involving human participants were reviewed and approved by Local ethic committee, CPP Paris VI. The patients/participants provided their written informed consent to participate in this study. Written informed consent was obtained from the individual(s) for the publication of any potentially identifiable images or data included in this article.

## Author contributions

VF, SW, and VN conceived and designed the study and interpreted the data. VF identified, selected, and analyzed the different electrophysiological patterns, and performed the clinico-anatomical analyses. SW designed and performed the quantitative time-signal analyses, spike analyses, and statistics. KL collected, organized, and preprocessed the physiological data. PY wrote the software for spike sorting and provided advice on the analysis. J-DL wrote the software for manual annotations for our analyses. VF and DH planned the trajectories of the macro–microelectrodes and their anatomical targets. BM performed the surgical implantations. CA, VF, DH, VL, and VN performed the clinical evaluations. VF and SW wrote the initial draft of the manuscript. VF, SW, VL, and VN provided critical revisions to the manuscript. All authors contributed to the final version of the manuscript. All authors contributed to the article and approved the submitted version.

## Funding

This study was supported by the program Investissements d'avenir ANR-10-IAIHU-06 and grants from the OCIRP-ICM and the Fondation de l'APHP pour la Recherche - Marie-Laure PLV Merchandising. Marie-Laure PLV Merchandising was not involved in the study design, collection, analysis, interpretation of data, the writing of this article or the decision to submit it for publication.

## Conflict of interest

The authors declare that the research was conducted in the absence of any commercial or financial relationships that could be construed as a potential conflict of interest.

## Publisher's note

All claims expressed in this article are solely those of the authors and do not necessarily represent those of their affiliated organizations, or those of the publisher, the editors and the reviewers. Any product that may be evaluated in this article, or claim that may be made by its manufacturer, is not guaranteed or endorsed by the publisher.

## Supplementary material

Supplementary Figure 1 *Example of multichannel data showing three patterns within a single time period*.

Supplementary Figure 2 *Correlation LFP between micro- and macro-contacts*. For each Nodule and each pattern, the microelectrode LFP is correlated with each macro-contact of the same shaft (1 = closest to microelectrode, 8 = farthest from microelectrode). Macro-contacts were bipolar referenced. The numbers and size of markers denote positive (yellow) and negative (blue) correlation values. Note the largest correlation with the closest macro-contact, as well as the flip of correlation values in the next contact, showing that the patterns were highly localized to the tip of the shaft.

Supplementary Figure 3 Representative SEEG recordings obtained from the three patients during the clinical evaluation.
