# Supplementary figures and images for: Human periventricular nodular heterotopia shows several interictal epileptic patterns and hyperexcitability of neuronal firing
Source: Front Neurol. 2022 Nov 11;13:1022768. doi: 10.3389/fneur.2022.1022768 (PMC9695411; doi:10.3389/fneur.2022.1022768)

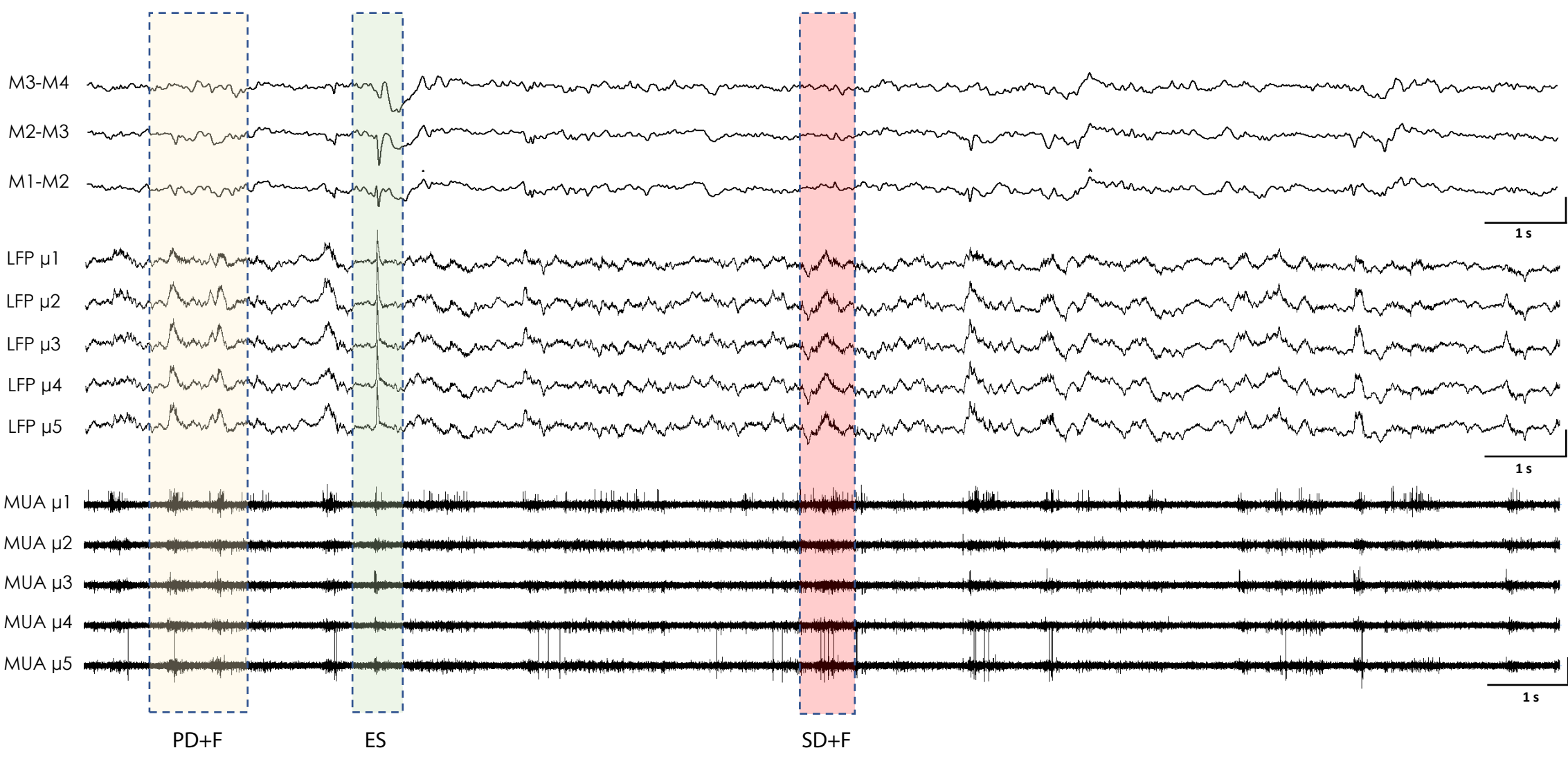

Supplement: Supplementary Figure 1 — Example of multichannel data showing three patterns within a single time period. [file Data_Sheet_1.PDF]

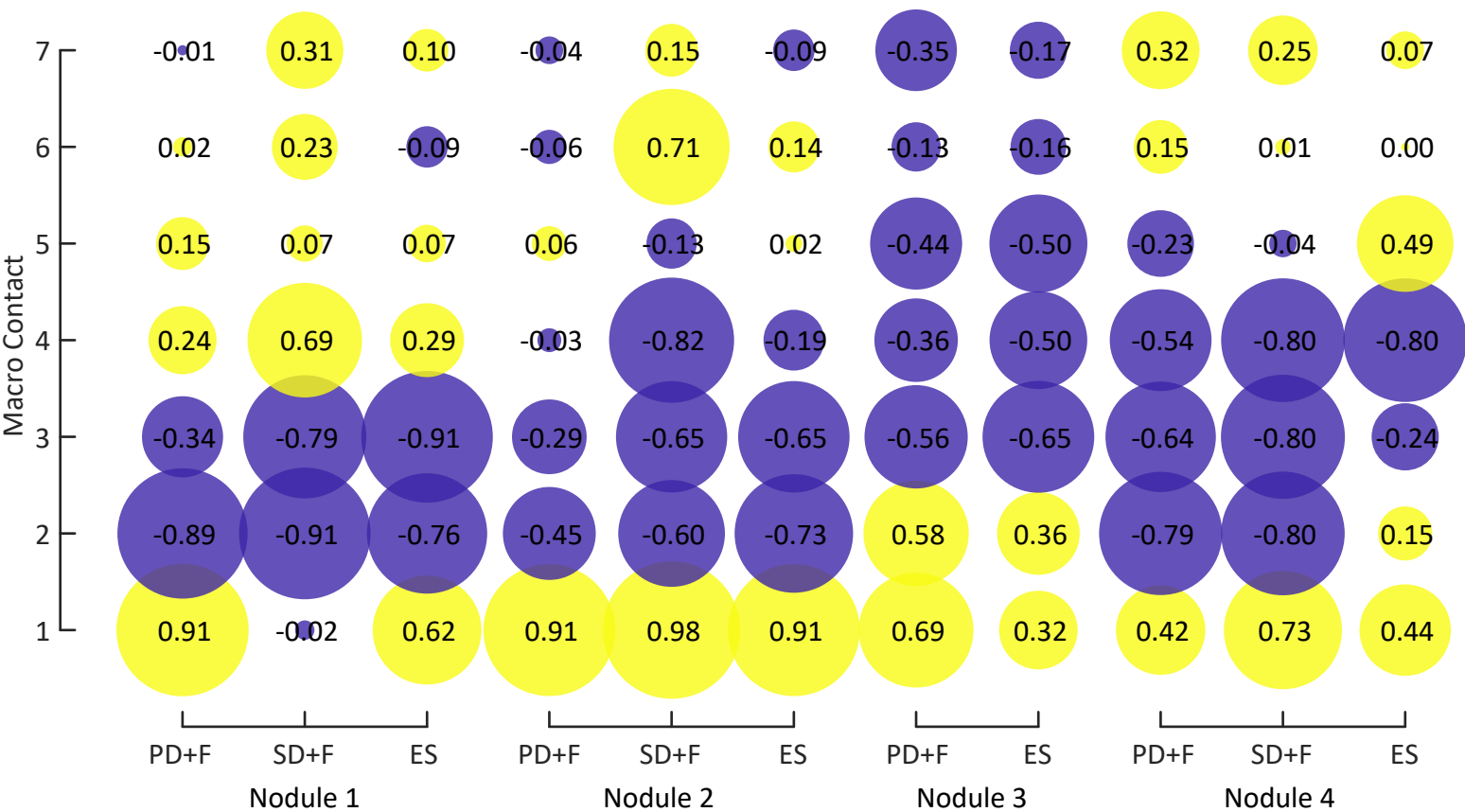

Supplement: Supplementary Figure 2 — Correlation LFP between micro- and macro-contacts. For each Nodule and each pattern, the microelectrode LFP is correlated with each macro-contact of the same shaft (1 = closest to microelectrode, 8 = farthest from microelectrode). Macro-contacts were bipolar referenced. The numbers and size of markers denote positive (yellow) and negative (blue) correlation values. Note the largest correlation with the closest macro-contact, as well as the flip of correlation values in the next contact, showing that the patterns were highly localized to the tip of the shaft. [file Data_Sheet_2.PDF]
